# Prospects of HA-Based Universal Influenza Vaccine

**DOI:** 10.1155/2015/414637

**Published:** 2015-02-15

**Authors:** Anwar M. Hashem

**Affiliations:** ^1^Department of Medical Microbiology and Parasitology, Faculty of Medicine, King Abdulaziz University, P.O. Box 80205, Jeddah 21589, Saudi Arabia; ^2^Special Infectious Agents Unit, King Fahd Medical Research Center, King Abdulaziz University, P.O. Box 21589, Jeddah 21589, Saudi Arabia

## Abstract

Current influenza vaccines afford substantial protection in humans by inducing strain-specific neutralizing antibodies (Abs). Most of these Abs target highly variable immunodominant epitopes in the globular domain of the viral hemagglutinin (HA). Therefore, current vaccines may not be able to induce heterosubtypic immunity against the divergent influenza subtypes. The identification of broadly neutralizing Abs (BnAbs) against influenza HA using recent technological advancements in antibody libraries, hybridoma, and isolation of single Ab-secreting plasma cells has increased the interest in developing a universal influenza vaccine as it could provide life-long protection. While these BnAbs can serve as a source for passive immunotherapy, their identification represents an important step towards the design of such a universal vaccine. This review describes the recent advances and approaches used in the development of universal influenza vaccine based on highly conserved HA regions identified by BnAbs.

## 1. Introduction

Influenza viruses cause highly contagious respiratory tract infections associated with high morbidity and mortality rates. Complications, hospitalization, and associated death most directly impact young children, individuals with chronic diseases, and the elderly [1]. Each year, seasonal influenza epidemics affect up to 500 million people, causing 3 to 5 million cases of severe illness, death of up to 500,000 people, and debilitating economic costs worldwide [2].

All influenza viruses (A, B, and C) belong to* Orthomyxoviridae* family. Among these genera, influenza types A and B viruses are associated with severe respiratory infections in humans. Influenza A viruses are categorized into different subtypes based on the surface hemagglutinin (HA) and neuraminidase (NA) glycoproteins. To date, there are at least 18 HA (1–18) and 11 NA (1–11) subtypes including the recently isolated highly divergent influenza A viruses from bats (H17N10 and H18N11) [3, 4]. On the other hand, influenza B viruses have diverged into two antigenically distinct lineages, Yamagata and Victoria [5].

Influenza A viruses infect many animal species including humans, pigs, horses, dogs, cats, sea mammals, and birds, while influenza B viruses are mainly restricted to humans [6, 7]. Most combinations of influenza A HA and NA subtypes have been isolated from aquatic birds (except for H17N10 and H18N11 from bats), which serve as a natural reservoir for influenza A viruses [7–9]. These viruses in wild aquatic birds are usually benign and evolutionarily stable, but they are in continuous evolution in mammalian hosts and land-based poultry [10, 11]. The evolution rate of influenza A viruses in humans differs among the different segments with the surface proteins, especially HA, evolving faster than the internal proteins mostly due to the selective immune pressure imposed by the host's immune system as well as the structural restrictions on the internal proteins [8]. The gradual accumulation of point mutations in influenza genes especially those encoding HA and NA (antigenic drift), can lead to selection and emergence of novel variant strains which can cause annual epidemics [12]. In addition, antigenically novel strains or subtypes of influenza A virus can emerge and spread rapidly due to a major antigenic change known as antigenic shift, causing global pandemics such as the ones that occurred in the last century or the recent H1N1 pandemic (pdmH1N1) in 2009 [13–18].

Until 1997, only H1N1, H2N2, and H3N2 subtypes circulated in humans with limited cases of direct transmission of avian viruses to humans. It was believed that the differences in receptor specificity between human and avian viruses represent a host range barrier. However, since 1997, direct transmission of the highly pathogenic avian influenza (HPAI) H5N1 virus from poultry to humans has increased and resulted in high mortality rate [19]. Other avian viruses such as H9N2 [20], H7N7 [21], and H7N9 [22] have also been isolated from humans. Although human-to-human transmission of these viruses has been limited so far, the ability of these HPAI viruses to infect humans and cause disease as well as their persistent circulation in domestic poultry have raised the concerns about their potential to cause devastating pandemics.

## 2. Current Influenza Vaccines

Several vaccination strategies have been evaluated for prevention against influenza; however, inactivated vaccines (i.e., whole inactivated virus, split vaccine, or subunit vaccine) are the most widely used approaches [23]. More recently, live-attenuated influenza vaccine (LAIV) has been approved for use in Russia, Europe, and USA [24–27]. These vaccines are typically trivalent containing two influenza A strains (H1N1 and H3N2) and one influenza B strain [1]. Recently, a quadrivalent influenza vaccine containing two influenza B strains from both Yamagata and Victoria lineages in addition to the two influenza A strains was approved for use in the USA and Europe [27, 28].

These vaccines provide substantial protection by predominantly inducing HA and NA strain-specific neutralizing antibodies (Abs) [29, 30]. LAIV are usually more effective in eliciting broad immune response including mucosal, systemic, and cell-mediated responses compared to inactivated vaccines which are weak in inducing mucosal immunity [31]. Many factors can influence the efficacy of inactivated vaccines including the antigenic match between circulating and vaccine strains, the age of the recipients, and their history of influenza exposure [32]. When the vaccine and circulating viruses are antigenically matched, these inactivated vaccines show 70–90% efficacy in healthy adults aged <65 years. Effectiveness against culture-confirmed influenza illness among children aged >6 months to 18 years varies between 50 and 90% depending on their age. However, it is 20–70% effective in preventing hospitalization in the elderly [1]. On the other hand, LAIV was reported to have up to 93% overall efficacy against culture-confirmed influenza and 86% against a mismatched H3N2 strain [31–33]. LAIV was shown to be more protective than inactivated vaccines in children 6 months to 18 years of age [32]. Both vaccines have similar efficacy in individuals between 17 and 49 years of age [1]. However, in the elderly, both vaccines have reduced immunogenicity and efficacy. Thus, a combination of both might be required to increase vaccine efficacy [32].

## 3. Drawbacks of Current Influenza Vaccines

The high antigenic variability of HA and NA as well as the uncertainty about the actual circulating strains requires annual reformulation of seasonal vaccines to ensure strain match and to achieve sufficient protection of the population against this changing threat [23, 30, 34]. Production of seasonal influenza vaccines relies on global influenza surveillance programs and the use of strains recommended by the World Health Organization (WHO) 9 to 12 months ahead of the targeted season [35]. However, vaccine manufacturing, testing for effectiveness, approval by regulatory authorities, and distribution are slow processes [36], which in addition to the short shelf life of these vaccines [37] can render the vaccine fairly ineffective. Furthermore, complex egg adaptation and growth characteristics required for some viruses such as the HPAI H5N1 viruses [34] might further delay vaccine production. Most importantly, any mismatch between the strains selected for the vaccine and those circulating due to either inaccuracy of prediction or introduction of a completely new strain during this lengthy production period might result in reduced efficacy and could be potentially devastating [38–43]. As witnessed in the 2009 pdmH1N1 outbreak, completely new strains can unexpectedly emerge. Spread of new pandemic strains is difficult to contain because of the time required to engineer and manufacture effective vaccines and to prepare reagents required for vaccine lot release. These limitations clearly highlight the importance of developing a universal influenza vaccine.

## 4. The Viral Hemagglutinin

HA is a classical type I membrane glycoprotein which functions as a sialic acid binding and membrane fusion protein during virus entry into target cells [44]. X-ray crystallographic studies show the HA molecule as a homotrimer in its neutral pH form which projects from the viral envelope to form a rod-shaped structure [45]. Each monomer in this trimer is initially synthesized as a single polypeptide precursor (HA0) in infected cells, which is later cleaved by host trypsin-like proteases into two subunits, HA1 and HA2, linked by a single disulfide bond [44]. The HA1 subunit forms a membrane-distal globular head that contains the receptor-binding site (RBS) and most of the antigenic regions recognized by neutralizing Abs [46–48]. Five antigenic sites have been described in the head domain of the H1 subtype: Sa, Sb, Ca1, Ca2, and Cb. These sites are designated as A, B, C, D, and E in the H3 subtype [49, 50]. On the other hand, HA2 forms most of the stem-like structures that anchor the globular domain to the viral membrane [51].

Cleavage of precursor HA0 by host proteases is a prerequisite for virus infectivity [52] and a crucial determinant of virulence and tissue tropism [53–55]. The cleavage site is a predominant surface loop near a deep cavity in HA0 [45]. This cleavage event results in structural rearrangements in which the nonpolar N-terminus amino acids of HA2 (the fusion peptide) are repositioned to the interior of the trimer, thereby burying the ionizable residues in the cleaved HA and generating a fusion competent structure [45]. Upon acidification in the endosome, HA undergoes irreversible conformational changes that result in extrusion of the HA2 N-terminal fusion peptide domain from its buried position to the end of a long coiled-coil domain. This allows its interaction with the target membrane and ultimately results in membrane fusion and release of the viral RNA segments into the cytoplasm [45, 56].

## 5. Identification of Broadly Neutralizing Anti-HA Abs (BnAbs)

**Figure 1:**
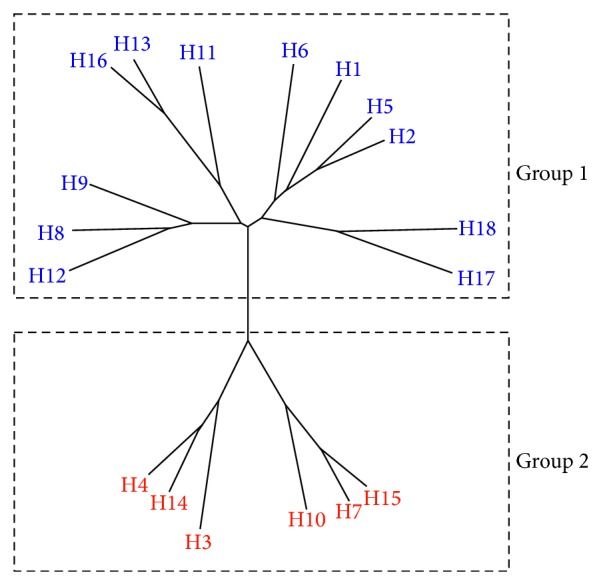
Phylogenetic tree of the 18 influenza A virus subtypes, classified into two groups: Group 1 and Group 2. Representative HA protein sequences were selected for each subtype from viruses belonging to following subtypes (H1N1, H2N2, H3N2, H4N6, H5N1, H6N2, H7N3, H8N4, H9N2, H10N7, H11N6, H12N5, H13N6, H14N5, H15N9, H16N3, H17N10, and H18N11). The phylogenetic tree was made with Geneious version 7.0.

The HA protein represents an attractive target for vaccine development because of its important roles in the early stages of virus infection. However, the host immune system usually recognizes the bulky and highly variable-immunodominant globular head domains in the HA which shield the more conserved regions such as those in the stem part [57, 58]. In addition, memory immune response is usually elicited or recalled against these immunodominant epitopes from previously encountered strain “original antigenic sin.” Therefore, development of BnAbs against diverse viral strains could be challenging as influenza A HA varies among not only the different 18 subtypes (H1–H18) which fall into two distinct phylogenetic groups (Figure 1) but also among the different strains within each subtype. Nonetheless, several groups have isolated Abs with broad inhibitory spectrum which bind to highly conserved epitopes in diverse influenza viruses from Group 1, Group 2, or both groups of the HA protein using naïve or immune phage display Ab libraries, Ab cloning from sorted plasmablasts and plasma cells, or hybridomas of memory B cells. Importantly, most of these studies have shown that cross-subtype BnAbs can be induced upon vaccination or infection in humans and animals which raises the prospects of HA-targeted universal influenza vaccine development.

### 5.1. HA Stem BnAbs

The first BnAb against influenza, C179 mAb, was isolated from mice immunized with H2N2 vaccine. This Ab neutralized several strains from Group 1 HA viruses including H1, H2, H5, and H9 viruses by preventing the low pH-dependent HA conformational change and the subsequent membrane fusion [59–63]. More recently, several human BnAbs were isolated including F10 and CR6261 Abs from human Ab phage display libraries prepared from nonimmune naïve and memory B cells from seasonally vaccinated individuals, respectively [64–67]. Both Abs were encoded by the germline V_H_1–69 genes and were cross-reactive and inhibitory against several Group 1 HA viruses but not viruses from Group 2* in vitro* and* in vivo*. F10 and CR6261 inhibited low-pH-induced HA conformational change and syncytia formation by targeting a conformational pocket-like epitope in the membrane-proximal stem of HA1/HA2 formed by the fusion peptide and the HA2 helix A [64–67]. Other human BnAbs against Group 1 HA viruses have also been isolated from B cell hybridomas from individuals vaccinated with seasonal vaccines. Abs such as FB110, FE43, and FC41 were found to recognize an acid-sensitive epitopes in the stem region and prevent the cleavage of immature HA0 into HA1 and HA2 [68]. Similarly, data on PN-SIA49 Ab suggest that its epitope is in the stem region but close to the HA globular head [69–71]. Human Abs with less cross-reactivity extent against the stem of Group 1 HA viruses were also obtained from plasmablast of pdmH1N1 infected individuals [72], bone marrow of H5N1 survivors [73, 74], and plasmablast of adults vaccinated with subunit pdmH1N1 vaccine [75] (Table 1).

While numbers are yet limited compared to Group 1 Abs, several BnAbs against Group 2 HA viruses were also isolated using hybridomas of peripheral memory B cells from individuals vaccinated with seasonal vaccine. Interestingly, CR8020 and CR8043 were obtained from the same individual but used different V_H_ genes families (1–18 and 1–3, resp.) [76, 77]. These two BnAbs target a conserved epitope in the HA stem base of Group 2 HA viruses (H3, H7, and H10) which is distinct from those recognized by Group 1 Abs. The epitopes for both CR8020 and CR8043 overlap in the stem base *β* sheet and fusion peptide, but the two Abs use different approach angles with different contact residues [76, 77]. Both Abs prevented immature HA0 cleavage into HA1 and HA2 and stabilized the perfusion HA conformation [76, 77] (Table 1).

Wider range BnAbs targeting epitopes shared by both Groups 1 and 2 subtypes were also described. F16v3 is a good example of these Abs in which it was isolated and cloned from a single CD138^+^ plasma cell obtained from a pdmH1N1 infected individual and vaccinated with seasonal vaccine. F16v3 bound to all 16 HA subtypes and neutralized H1, H3, H5, and H7 viruses* in vitro* and* in vivo*. It bound to a conserved epitope in the stem similar to CR6261 and F10, using different approach by avoiding contact with nearby glycan in Group 2 viruses which extended its breadth to both groups. Interestingly, its* in vivo* efficacy was found to be dependent on antibody effector mechanisms [78]. Similar Abs targeting similar epitopes in both HA groups and encoded by the same germline V_H_3–30 genes were also reported by others upon seasonal vaccination such as 39.29, 81.39, and PN-SIA28 Abs [69, 70, 79, 80] (Table 1).

CR9114 sets itself as a unique BnAb as it neutralizes not only the two groups of influenza A viruses but also the two lineages of B viruses. This unique Ab was obtained from a phage display Ab library prepared from an individual vaccinated with seasonal influenza vaccine and used germline V_H_1–69 genes. It bound to the F subdomain and neutralized Group 1 and Group 2 influenza A viruses* in vitro* and* in vivo* by blocking the pH-dependent conformational rearrangement required for membrane fusion similar to F16v3 Ab. Interestingly, it failed to neutralize influenza B viruses* in vitro* but protected mice from lethal challenges with viruses from both Victoria and Yamagata lineages [81] (Table 1).

Notably, many of the stem-targeting BnAbs were preferentially encoded by V_H_1–69 germline genes, showed no hemagglutination inhibition (HI) activity, and competed with C179 mAb. They bind to a highly conserved region in the HA stem region and share the fusion peptide as a part of their epitopes. The mechanism of cross-neutralization by these Abs depends on stabilizing the HA perfusion conformation or preventing the pH-dependent HA conformational changes required for the membrane fusion step.

### 5.2. HA Head BnAbs

Several subtype-specific BnAbs targeting epitopes in the head domains of wide range of strains belonging to H1, H2, H3, and H5 subtypes have been isolated using several approaches (Table 2). H1-specific BnAbs such as 1F1, 2D1, 5J8, CH65, and CH67 were isolated from healthy elderly or adults, pdmH1N1 infected individuals, or people vaccinated with seasonal influenza vaccines. These Abs prevented viral attachment and sometimes viral release by recognizing conserved epitopes in the antigenic sites in the head domain encompassing or near the RBS [72, 82–89]. Several H2- or H3-specific BnAbs were also obtained from healthy volunteers and found to have HI and neutralization activity by binding to similar epitopes [90–94]. Of note, F005-126 Ab showed no HI activity and mediated its neutralization by preventing the low-pH-induced conformational change in HA similar to stem-targeting Abs, although it bound to the head domain in H3 viruses [94]. Similarly, anti-head domain BnAbs targeting conserved linear or conformational nonlinear epitopes encompassing or in close proximity to the RBS in several H5 clades have been isolated from H5N1 survivors. These H5-specific BnAbs neutralized several H5 viruses* in vitro* and* in vivo* and showed HI activity [95–97]. BnAbs targeting epitopes in the head domain of influenza B viruses only have also been found [81]. Here, CR8033 showed HI activity against viruses from Yamagata lineage and mediated its neutralization by interfering with receptor binding. On the other hand, it had no HI activity against strains from Victoria lineage and it neutralized these viruses by preventing viral release from infected cells in a similar fashion to CR8071 which neutralizes both lineages by inhibiting viral release [81].

S139/1 is a murine mAb isolated from mice immunized intranasally with H3 virus and recognizes a novel conformational epitope near the RBS. It neutralized viruses from Group 1 and Group 2 by means of avidity [98, 99]. The human C05 Ab also neutralized viruses from both groups* in vitro* and protected mice against lethal H1N1 and H3N2 challenges. Crystal structure data suggest that C05 prevents viral attachment through steric hindrance and cross-linking of HA on the surface of the virus via avidity similar to S139/1 [67]. Several other BnAbs targeting sites in the HA globular head near the RBS in Group 1 and/or Group 2 viruses such as FE17 and 2G1 were also identified from vaccinated or healthy donors [68, 90, 91, 93].

Compared to stem-targeting BnAbs which block viral fusion and require Fc-IgG Fc receptors (Fc*γ* Rs) interaction to confer protection via effector mechanisms such as antibody dependent cellular cytotoxicity (ADCC) [78, 100], most anti-head Abs neutralize viruses by inhibiting viral attachment to sialic acids receptors on target cells. Furthermore, while most subtype-specific BnAbs neutralize several strains within each subtype by targeting conserved elements in the head domain, their breadth and cross-reactivity are limited to one subtype most likely due to their interaction with highly variable residues in HA head domain. On the other hand, broader spectrum anti-HA head Abs, such as S139/1 and C05, avoid contact with such residues. Nonetheless, isolation of these BnAbs indicates that some regions in the head domain are sufficiently conserved and exposed to induce broader protection than initially thought.

## 6. Induction of BnAbs

Several studies have shown that BnAbs targeting conserved HA stem or head domains can be induced upon seasonal influenza infection [67, 92], H5N1 infection [73, 95, 96], and pdmH1N1 infection [72, 78, 101–104] in humans. Several other reports have also shown that seasonal or pdmH1N1 vaccines can induce such Abs against diverse viruses [65, 68, 75, 76, 81, 88]. Interestingly, very low levels of BnAbs against Group 1 and 2 viruses including viruses that have never been experienced in humans have been found in intravenous immunoglobulin (IVIG) and prevaccination serum samples [64]. Moreover, even though many of these BnAbs were constructed from combinatorial Ab libraries, naturally occurring BnAbs have been isolated [93]. These Abs have been shown to be long-lasting and to have high degree of somatic hypermutation suggesting their memory origin [68, 75, 102, 104]. Current immunological and technological advances allowed the generation of innovative and novel vaccine platforms to elicit heterosubtypic protection. These advances include use of adjuvants, alternative delivery vectors, routes or regimens, and novel HA-based vaccines.

### 6.1. Use of Adjuvants

Coadministration of adjuvants is an effective approach for inducing cross-protection. Toll-like receptors (TLRs) were proven to be highly effective in inducing potent immune response. For example, flagellin, which is a TLR-5 agonist, has been used in several studies and shown to enhance immune responses and cross-protection when used as an adjuvant in virus-like particles (VLPs) expressing influenza HA or when it is fused to HA [75, 105]. Other TLR ligands such as the TLR-3 ligand (ligand polyinosinic-polycytidylic acid, poly I:C) [106] and the licensed Adjuvant System 04 (AS04) which is comprised of TLR-4 agonist (3-O-desacyl-4-monophosphoryl lipid A, MPL) and aluminum hydroxide (Alum) [107] have also been shown to enhance heterosubtypic protection. Interestingly, a recent report has demonstrated the importance of TLR-7 in generating cross-protection upon immunization with inactivated H5N1 vaccine in mice, suggesting that the use of TLR-7 ligands can provide a way to enhance heterosubtypic protection [108]. Other adjuvants have also been shown to boost Ab response and induce anti-stem BnAbs, but further studies are clearly needed [109, 110].

Targeting HA protein to antigen presenting cells (APCs) can also represent a promising approach to enhance cross-protective Ab response. For instance, HA2 segment (residues 23–185) inserted into a detoxified adenylate cyclase toxoid (CyaA-E5), denoted as CyaA-E5-HA2, induced potent T cell responses, broadly cross-protective HA2-specific Abs, and protected mice from lethal homologous and heterologous viruses [111]. Also, we recently showed that intranasal immunization of mice with recombinant adenovirus (rAd) expressing fusion protein consisting of codon-optimized HA2-subunit of A/California/7/2009(H1N1) virus fused to a trimerized form of CD40L completely protected mice against lethal challenges with divergent influenza A subtypes including H1N1, H3N2, and H9N2. Importantly, use of CD40L as targeting molecule and molecular adjuvant with HA2 subunit in this study elicited BnAbs capable of neutralizing 13 subtypes of influenza A viruses* in vitro* [112]. Thus, the use of such adjuvants or approaches can overcome the weak immunogenicity of highly conserved regions and could induce BnAbs.

### 6.2. Alternative Delivery Vectors, Routes, or Regimens

Recombinant replication-deficient or live-attenuated vectors (Reviewed in [113]) or DNA vaccines have been shown to be promising delivery candidates for inducing long-lasting and broad immunity against influenza. For instance, immunization with rAd or DNA vaccine expressing synthetic consensus HA protein from H1 viruses (rAd-HA1-con) [114], H7 viruses (pH7HA) [115], or H5 viruses (pCHA5) [116] induced potent Ab responses and protected mice against diverse lethal subtype-specific challenges, suggesting that consensus HA proteins can at least provide subtype-specific immunity. Other platforms such as VLP have also been used and shown to be effective in inducing broad immunity [105, 117].

Several studies have also shown induction of heterosubtypic immunity by alternative immunization routs or regimens. Intranasal immunization with monovalent replication-deficient delNS1-H1N1 influenza virus vaccine elicited significant levels of circulatory IgG and local IgA in humans. Importantly, mucosal Abs showed heterosubtypic neutralization against H3N2 and H5N1 viruses [118]. Oral vaccination of mice with inactivated whole influenza virus (A/PR8/34) induced high levels of mucosal and circulatory heterosubtypic IgG and IgA Abs with HI activity. This response protected mice completely against homologous or heterologous challenges and partially against heterosubtypic virus [119]. Prime-boost regimen with DNA vaccine expressing H1 and seasonal vaccine or rAd expressing H1 induced anti-stem BnAbs and conferred complete protection against divergent H1N1 viruses in mice, ferrets, and nonhuman primates [120].

### 6.3. Novel HA Vaccines

Several novel vaccines or immunogens based on the isolation of BnAbs, structural data obtained from BnAbs studies, or the identification of highly conserved regions have been developed and examined. While most of these novel vaccines provided limited heterosubtypic protection against lethal influenza challenge, they represent an important step towards universal influenza vaccine development.

#### 6.3.1. Peptide-Based Vaccines

Immunization of mice with synthetic peptide (amino acids 76–130) from the long *α*-helix of H3 HA2 subunit (strain A/HK/68 HA) fused to keyhole limpet hemocyanin (KLH) not only improved mouse survival and reduced viral load against homologous challenge but also provided partial protection against heterosubtypic viruses from Group 1 HA [121]. Use of VLP expressing 180 copies of the 20-residue A-helix from HA2 in Group 1 viruses which is a major part of the epitope recognized by CR6261 and F10 BnAbs induced cross-reactive Abs against Group 1 but not Group 2 viruses. However, these VLPs failed to protect mice and the elicited Abs showed no neutralization activity* in vitro* [117]. Through comprehensive bioinformatics analysis of all publicly available HA sequences, we recently showed that the N-terminal 14 amino acids of the fusion peptide (GLFGAIAGFIEGGW) represent the most conserved peptide in all 16 subtypes of influenza A and the two genetic lineages of influenza B viruses [122–125]. Immunization of rabbits with this peptide linked to KLH generated universal anti-influenza Abs (Uni-1 Abs) that are cross-reactive to virtually all influenza HA subtypes with high specificity [122, 123]. Importantly, Uni-Abs cross-neutralized multiple subtypes of influenza A virus by inhibiting the pH-dependent viral and cellular membranes fusion [124]. Interestingly, at least two similar neutralizing Abs targeting the fusion peptide were generated from immunization of mice with recombinant H5N1 HA0 [126] or memory B cells of pdmH1N1 infected person [127], suggesting that this highly conserved liner peptide is at least partially exposed to induce Abs and is accessible for binding which warrants further investigation.

#### 6.3.2. Conserved HA Regions-Focused Vaccines

Despite the efficacy of anti-head BnAbs, these Abs are usually more efficient in selecting escape mutants* in vitro* and* in vivo* compared to anti-stem Abs which target highly conserved epitopes across divergent influenza subtypes [68, 128, 129]. Furthermore, isolation of anti-stem BnAbs from pdmH1N1 infected or vaccinated individuals suggest that vaccines containing HA head domains with substantial difference compared to seasonal strains can skew the immune response and boost HA stem Abs by stimulating the rare memory B cells targeting the highly conserved sequences in the HA protein [101–104]. Therefore, it was proposed that vaccine aimed at eliciting stem BnAbs could be a good way to provide a long-lasting universal protection against influenza. Several approaches including the use of “headless” HA protein, HA in the neutral-pH conformation, masking of highly variable-immunodominant regions, or chimeric HA proteins have been used.

For example, headless HA protein lacking the globular domain from H1N1, H2N2, or H3N2 viruses and expressed as recombinant protein or by VLPs was developed and investigated. However, while it elicited anti-HA stalk Abs, its protection was restricted to viruses whose HAs are in the same phylogenetic group as that of the immunogen [58, 130]. Similar headless H5 recombinant vaccine produced in baculovirus has been developed and is being investigated [131]. In another approach, immunization of mice with recombinant HA2 subunit with regions (7–46) and (290–321) from HA1 of H3N2 virus expressed in its neutral pH-conformation protected mice against homologous challenge only and elicited neutralizing Abs against other strains within the same subtype [132].

Other interesting approaches such as glycan masking and chimeric HA protein have been tested to skew immune response towards conserved HA regions. For instance, Eggink et al. have shown that intramuscular immunization of mice with recombinant hyperglycosylated HA protein, glycans introduced in 7 N-linked glycosylation sites at the antigenic sites in the head domain to shield these immunodominant sites, adjuvanted with poly(I:C), can skew Ab response towards the stem region by up to 9 folds after 3 immunizations, enhance heterologous protection, and elicit heterologous and heterosubtypic Ab response [133]. Similarly, glycan masking of H5N1 HA protein has resulted in induction of BnAbs similar to CR6162 and showed cross-clade protection against heterologous H5N1 viruses [134]. Other groups have also shown that prime-boost immunization with vaccines expressing chimeric HA proteins comprised of the globular head domain of unrelated HA subtypes with the stalk domain of particular subtypes (such as cH4/3, cH5/3 or cH7/1 HAs) can skew the Ab response towards the stem, induce highly cross-reactive anti-stem BnAbs to heterologous HAs, and confer broad cross-protection against various viruses [135–137].

#### 6.3.3. Polyvalent HA Vaccines

Vaccines expressing multivalent or combination of HA have also been investigated to evaluate their abilities to confer heterologous and heterosubtypic protection. Trivalent recombinant H5 vaccine with oil-in-water emulsion adjuvant SP01 provided complete cross-protection against HPAI H5N1 virus in mice compared to monovalent vaccine [138]. Similar results were also observed upon trivalent or tricalde H5 DNA immunization against several heterologous H5 clades and subclades [139–141]. However, the breadth was dependent on the choice of the HA in the vaccine [139]. Immunization with rAd vectors expressing combination of HA from H1, H5, H7, and H9 induced high levels of stem-specific neutralizing Abs and conferred protection against virus replication following challenge with antigenically distinct viruses from these subtypes [142]. Furthermore, recombinant modified vaccinia virus Ankara (MVA) vector expressing HA from H5N1 A/Vietnam/1203/04, A/Indonesia/669/06, and A/Anhui/01/05 viruses (MVAtor-tri-HA vector) elicited potent cross-neutralizing protection in mice against H5N1 challenge from divergent clade. Importantly, it induced cross-clade neutralizing immunity against twenty different H5N1 strains from six clades in guinea pigs [143].

## 7. Conclusion and Future Directions

One of the major inherent drawbacks of current influenza vaccines is the need for annual reformulation to antigenically match circulating strains. This is mostly due to the inability of these vaccines to induce cross-protective immune responses against the highly diverse influenza viruses. Isolation of arsenal of anti-HA BnAbs and the elucidation of the structural, molecular, and biological basis of their neutralizing and protective abilities helped in the development of several HA-based universal vaccine candidates. While the efficacy of many of the aforementioned novel approaches based on rational vaccine design and the use of effective adjuvants and alternative immunization regimens or routes seem to be limited to certain influenza subtypes or groups, they clearly show an improvement over the current annual vaccination strategies, demonstrate a wide range of options available for more exploration, and represent a promising step towards the development of broadly protective influenza vaccine. Nonetheless, development of an ideal HA-based universal influenza vaccine which could provide life-long protection against all influenza viruses clearly necessitates more preclinical and clinical studies to elucidate the underlying molecular mechanisms involved in eliciting such cross-protective immunity and more importantly to eliminate any safety concerns associated with these novel approaches.

## Figures and Tables

**Table 1:** Summary of anti-HA stem BnAbs.

Ab	Neutralization breadth^*^	Year^¶^	References
C179	Group 1 viruses	1993	[59–63]
F10	Group 1 viruses	2009	[64]
CR6261	Group 1 viruses	2008	[65–67]
FB110	Group 1 viruses	2010	[68]
FE43	Group 1 viruses	2010	[68]
FC41	Group 1 viruses	2010	[68]
PN-SIA49	Group 1 viruses	2009	[69–71]
70-5B03	Group 1 viruses	2011	[72]
70-1F02	Group 1 viruses	2011	[72]
1000-3D04	Group 1 viruses	2011	[72]
1009-3B05	Group 1 viruses	2011	[72]
1009-3E06	Group 1 viruses	2011	[72]
A06	Group 1 viruses	2008	[73, 74]
09-2A06	Group 1 viruses	2012	[75]
09-3A01	Group 1 viruses	2012	[75]

CR8020	Group 2 viruses	2011	[76, 77]
CR8043	Group 2 viruses	2014	[77]
12D1	Group 2 viruses	2010	[121]

F16v3	Group 1 and Group 2 viruses	2011	[78]
39.29	Group 1 and Group 2 viruses	2013	[80]
81.39	Group 1 and Group 2 viruses	2013	[80]
PN-SIA28	Group 1 and Group 2 viruses	2009	[69, 70, 79]
Uni-1	Group 1 and Group 2 viruses	2008	[122–125]

CR9114	Influenza A and B viruses	2012	[81]

^*^Neutralization breadth was based on *in vitro* neutralization and *in vivo* protection in cited references.

^¶^Year of first report.

**Table 2:** Summary of anti-HA head BnAbs.

Ab	Neutralization breadth^*^	Year^¶^	References
1F1	H1 subtype	2008	[82, 83]
2D1	H1 subtype	2008	[82, 84, 86]
CH65	H1 subtype	2011	[88]
CH67	H1 subtype	2011	[88, 89]
5J8	H1 subtype	2011	[85, 87]
1009-3B06	H1 subtype	2011	[72]
1009-3F01	H1 subtype	2011	[72]
1I20	H1 subtype	2008	[82, 83]
2B12	H1 subtype	2008	[82]
4D20	H1 subtype	2008	[82]

8F8	H2 subtype	2012	[90, 91]
8M2	H2 subtype	2012	[90, 91]

FabIF1A11	H3 subtype	2008	[92]
F005-126	H3 subtype	2011	[93, 94]

AVFluIgG01	H5 subtype	2009	[95]
AVFluIgG03	H5 subtype	2009	[95]
FLA3.14	H5 subtype	2007	[96]
FLD21.140	H5 subtype	2007	[96, 97]
FLD20.19	H5 subtype	2007	[96]
FLA5.10	H5 subtype	2007	[96, 97]

FE17	Group 1 viruses	2010	[68]

S139/1	Group 1 and Group 2 viruses	2009	[98, 99]
C05	Group 1 and Group 2 viruses	2012	[67]
2G1	Group 1 and Group 2 viruses	2012	[90, 91]
F045-092	Group 1 and Group 2 viruses	2011	[93]
F026-427	Group 1 and Group 2 viruses	2011	[93]

CR8033	Influenza A and B viruses	2012	[81]
CR8071	Influenza A and B viruses	2012	[81]

^*^Neutralization breadth was based on *in vitro* neutralization and *in vivo* protection in cited references.

^¶^Year of first report.
